# Is Dysplasia the Sole Determinant of the Prognosis in Oral Potentially Malignant Disorders?

**DOI:** 10.1111/jop.13617

**Published:** 2025-03-05

**Authors:** Pelin Güneri, Betul İlhan, Gaye Bolukbasi, Ali Veral, Joel B. Epstein

**Affiliations:** ^1^ Department of Oral and Maxillofacial Radiology Ege University School of Dentistry İzmir Türkiye; ^2^ Department of Pathology Ege University School of Medicine Bornova, İzmir Türkiye; ^3^ City of Hope National Medical Center Duarte California USA; ^4^ Cedars Sinai Health System Los Angeles California USA

**Keywords:** cancer progression, oral cancer, oral dysplasia

## Abstract

**Background:**

The dynamic nature of oral epithelial dysplasia, involving both progression and regression over time, complicates prognostic predictions for the lesion.

**Objective:**

This paper addresses the intricacies of this challenge and advocates for a nuanced strategy. It proposes a comprehensive assessment, tailored to individual patients, incorporating genetic, behavioral, and medical factors to enhance the precision of lesion evaluation.

1

During oral carcinogenesis, the mucosa progresses from normal epithelium to invasive carcinoma [1], with molecular and cytological alterations causing histological changes termed as “oral epithelial dysplasia (OED)”. OED is a histological marker of malignant transformation [1] and classified as mild, moderate, or severe based on architectural and cellular alterations [2]. These changes are dynamic and may stabilize, progress, or regress, particularly after lifestyle changes or cessation of cancer‐risk habits [3, 4, 5], although severe OED poses a higher risk of malignancy. Nevertheless, there are cases of non‐dysplastic lesions advancing to malignancy [1, 3], and histologically reported severe dysplasia exhibiting regression [6].

Before addressing the uncertain prognosis of OED, it is essential to examine contributing factors. Variability among pathologists in interpreting epithelial architectural and cytological features is a foremost limitation in the accuracy of dysplasia staging for predicting malignancy [5]. The current WHO grading system with its multi‐class approach further complicates OED staging [7]. The key criteria for staging include abnormal mitotic figures in the spinous and basal layers, mitosis in suprabasal layers, and disrupted epithelial cell polarity [4], but pathologists lack consensus on irregular stratification, basal cell polarity loss, nuclear size variation, atypical mitosis, and hyperchromatism [8]. To improve inter‐observer agreement, a binary classification system for OED is proposed to categorize lesions as “high‐risk” or “low‐risk” based on specific architectural and cytological changes [9], without further effectiveness than WHO's three‐tier grading system [10].

OED management includes surgical excision, medical treatments, cessation of risk factors (smoking, alcohol), and regular follow‐up [9]. Unfortunately, in a 5‐year follow‐up study, 28% of OED cases in 91 patients progressed to more severe dysplasia or malignancy; 69% showed remission or spontaneous resolution [11]. Contrary to existing literature, alcohol use and gender were not linked to lesion progression. Dysplasia severity ranked as the fourth strongest predictor of malignant transformation, with smoking status, lesion appearance, site, and size being the most significant factors [11].

Growing attention is being given to chronic inflammation identified as a potential cause [12] which involves cytokines, chemokines, prostaglandins, reactive oxygen species, and transcription factors, which can inhibit apoptosis, promote cell proliferation, and disrupt cell cycles and signaling pathways [13]. These mediators may contribute to immunosuppression and tumor progression by inducing epithelial‐mesenchymal transition and enabling invasion of lesions with genetic mutations in tumor suppressor and/or oncogenes [12].

The significance of host‐related factors such as demographics, health status, genomics, and lifestyle, which are equally important as microbial attributes, remains unclear. Given the impracticality of standardizing these varied factors, each study must be assessed within the specific context of its sample group. The literature manifests diverse outcomes influenced by methodological differences and host‐related variables. Alongside genetic and epigenetic influences, these factors can uniquely affect epithelial changes and OED prognosis in each patient.

The microenvironment's role during dysplastic transformation is strongly influenced by genetic and epigenetic factors, which can be partially modified through medical interventions like CRISPR or by improving lifestyle, health, and habits. Thus, predicting the malignant potential of a lesion is almost impossible when strict guidelines are employed. For oral potentially malignant disorders where initial biopsies fail to reveal OED, multiple biopsies can help capture the full lesion, reducing underdiagnosis. Correlating these biopsies with clinical data and examining deeper sections can significantly enhance diagnostic accuracy and provide a more comprehensive pathological assessment.

While certain aspects may not be strictly evidence‐based, the authors of this paper have outlined a proposed protocol based on best practices, particularly in instances where evidence is limited or unavailable, to support clinicians in effectively managing patients with OED:

- Clinical images of the lesion should be captured using an intraoral camera during each follow‐up. If an intraoral camera is unavailable, a mobile phone‐based camera can serve as an alternative.
- Considering “field cancerization,” vital staining solutions may be applied as oral rinses to detect recurrences or secondary malignancies.
- For stained areas, brush biopsy should be performed to collect cell samples for nuclear morphometric analysis. All specimens should be diagnosed as: (1) negative (no atypical cells), (2) suspicious (dyskaryotic cells or a few malignant cells), (3) positive (malignant cells), (4) insufficient (inadequate cell sample).
- Treatment choice (surgical or laser excision, cryosurgery, electrocautery, photodynamic therapy, topical or systemic medical treatment, and PUVA therapy) should be determined by the severity of dysplasia and follow‐up findings.
- A strict follow‐up regimen shall be conducted: mild dysplasia patients should be clinically monitored with the above‐mentioned procedures every 6 months, while moderate dysplasia patients shall be recalled every 3 months. Patients with severe dysplasia or carcinoma in situ require more frequent controls and should be followed up monthly. This routine should continue for 2 years, but lifelong monitoring is recommended due to the unpredictable nature of OED.

From a clinical point of view, we suggest that the degree of dysplasia should not be the sole criterion for determining treatment, particularly for “high‐risk” patients, even with mild or moderate OED. In such cases, aggressive treatment protocols including chemoprevention, photodynamic therapy, or immunotherapy may be warranted to prevent transformation [14]. The unpredictable prognosis of OED and the occurrence of malignancy without precursor lesions underscore the necessity for individualized monitoring and suggest that the severity of OED alone may not reliably determine the prognosis of an oral mucosal lesion [4, 15].

Given the complexity of OED development and prognosis, further research is needed to elucidate the factors driving disease progression and to develop more accurate prognostic tools and treatment strategies.

## Author Contributions

Conceptualization: Pelin Güneri, Betul İlhan, Joel B. Epstein. Data curation: Gaye Bolukbasi, Ali Veral, Betul İlhan. Supervision: Pelin Güneri, Betul İlhan. Writing – original draft: Pelin Güneri, Betul İlhan. Writing – review and editing: Pelin Güneri, Joel B. Epstein. All authors reviewed and approved the manuscript.

## Conflicts of Interest

The authors declare no conflicts of interest.

### Peer Review

The peer review history for this article is available at https://www.webofscience.com/api/gateway/wos/peer‐review/10.1111/jop.13617.

## Data Availability

The authors have nothing to report.
